# Generation Scotland: Donor DNA Databank; A control DNA resource

**DOI:** 10.1186/1471-2350-11-166

**Published:** 2010-11-23

**Authors:** Shona M Kerr, David CM Liewald, Archie Campbell, Kerrie Taylor, Sarah H Wild, David Newby, Marc Turner, David J Porteous

**Affiliations:** 1 Medical Genetics Section, Centre for Molecular Medicine, University of Edinburgh, Institute of Genetics and Molecular Medicine, Western General Hospital, Crewe Road, Edinburgh, UK; 2 Wellcome Trust Clinical Research Facility, University of Edinburgh, Western General Hospital, Crewe Road, Edinburgh, UK; 3 Scottish National Blood Transfusion Service, Royal Infirmary, Edinburgh, UK

## Abstract

**Background:**

Many medical disorders of public health importance are complex diseases caused by multiple genetic, environmental and lifestyle factors. Recent technological advances have made it possible to analyse the genetic variants that predispose to complex diseases. Reliable detection of these variants requires genome-wide association studies in sufficiently large numbers of cases and controls. This approach is often hampered by difficulties in collecting appropriate control samples. The Generation Scotland: Donor DNA Databank (GS:3D) aims to help solve this problem by providing a resource of control DNA and plasma samples accessible for research.

**Methods:**

GS:3D participants were recruited from volunteer blood donors attending Scottish National Blood Transfusion Service (SNBTS) clinics across Scotland. All participants gave full written consent for GS:3D to take spare blood from their normal donation. Participants also supplied demographic data by completing a short questionnaire.

**Results:**

Over five thousand complete sets of samples, data and consent forms were collected. DNA and plasma were extracted and stored. The data and samples were unlinked from their original SNBTS identifier number. The plasma, DNA and demographic data are available for research. New data obtained from analysis of the resource will be fed back to GS:3D and will be made available to other researchers as appropriate.

**Conclusions:**

Recruitment of blood donors is an efficient and cost-effective way of collecting thousands of control samples. Because the collection is large, subsets of controls can be selected, based on age range, gender, and ethnic or geographic origin. The GS:3D resource should reduce time and expense for investigators who would otherwise have had to recruit their own controls.

## Background

This paper describes the collection and initial characterisation of a resource of control DNA and plasma samples accessible for research, the Generation Scotland Donor DNA Databank (GS:3D). Common conditions such as cancer, cardiovascular disease, diabetes and mental illness create a heavy burden of morbidity and mortality in developed countries and are consequently of major public health importance [1,2]. These diseases have a significant heritable component but are difficult to analyse by traditional genetic techniques because they typically result from the combined effect of multiple genetic, lifestyle and environmental factors rather than from the effect of a single gene [2-4]. The completion of the Human Genome Project and the availability of cost-effective, high-throughput methods for systematically characterising genome-wide sequence variation have made it possible to dissect the genetic variants that predispose to complex diseases [2,3]. This is accomplished through genome-wide association studies in which genetic variants (such as single nucleotide polymorphisms or copy number variants) are typed across the whole genome in large numbers of cases and controls. If a statistically significant increase in the frequency of a variant is observed in cases compared to controls, the region of the genome in linkage disequilibrium with the variant is implicated in disease risk [2,3]. Genome-wide association studies have already yielded promising results for a number of common diseases [5,6], with wide-ranging implications for the future of healthcare including better understanding of disease mechanisms, more accurate diagnosis, and personalised therapy [2,7]. Nevertheless, reliable detection of disease-associated genetic variants requires very large sample and data sets and considerable associated infrastructure [5,7]. Generation Scotland is a multi-institution, cross-disciplinary collaboration which aims to create an ethically sound, family- and population-based resource for identifying the genetic basis of common complex diseases [8,9]. One of Generation Scotland's resources, the Generation Scotland: Donor DNA Databank (GS:3D), is a collection of 5,000 control DNA and plasma samples taken with full consent from blood donors across Scotland. This will help solve the problem of gathering the large numbers of controls that are essential for the success of case-control studies. GS:3D has also helped to develop an information technology infrastructure to support large-scale genetics research studies, including the implementation of systems to ensure efficient management of data and samples. GS:3D samples, together with non-identifiable demographic information, will be made available to researchers under appropriate standards of governance.

### The objectives of GS:3D were

1. To recruit and minimally phenotype a cohort of healthy control participants from across Scotland, facilitating studies aimed at identification of genetic variants relevant to common complex diseases.

2. To extend a technology platform of informatics in genetics research, including the creation of user-friendly databases of study data.

## Methods

### Recruitment of participants

**Figure 1:**
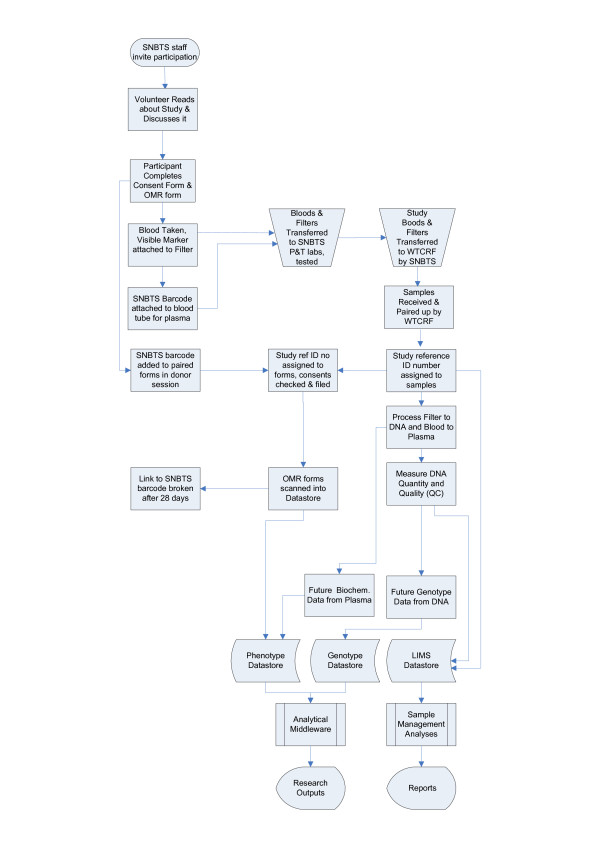
**GS:3D Schematic**. Schematic outline of the GS:3D study methodology.

The steps used to implement the GS:3D protocol are illustrated schematically in Figure 1. Participants were recruited through the donor services directorate of the Scottish National Blood Transfusion Service (SNBTS). Each donor undergoes a careful assessment to determine their eligibility to donate blood, requiring answers to a series of standard questions relating to their general health, lifestyle, past medical history and medication. They were eligible to participate in GS:3D if they were not first time donors, were aged between 17 and 70 years, and fulfilled the other clinical criteria for eligibility of blood donors. Most relevant among these clinical criteria for genetic research of common complex conditions are exclusion if an individual:

(1) has a history of angina, ulcerative colitis or Crohn's disease.

(2) is taking anticoagulant medication.

(3) is taking beta blockers to treat cardiovascular disease.

(4) has had cardiac surgery, a malignancy, a stroke or transient ischaemic attack.

(5) has ischaemic heart disease, dementia, inflammatory bowel disease, multiple sclerosis, narcolepsy or active rheumatoid arthritis.

(6) has diabetes insipidus or diabetes mellitus which requires medication.

(7) requires maintenance treatment for mental health problems.

Potential donors are also excluded if their haemoglobin levels are below a threshold (125 g/L females, 135 g/L males). Donors must weigh at least 7st 12lb (50 kg). Blood donors are therefore people in good general health, especially regarding cardiovascular health.

**Figure 2:**
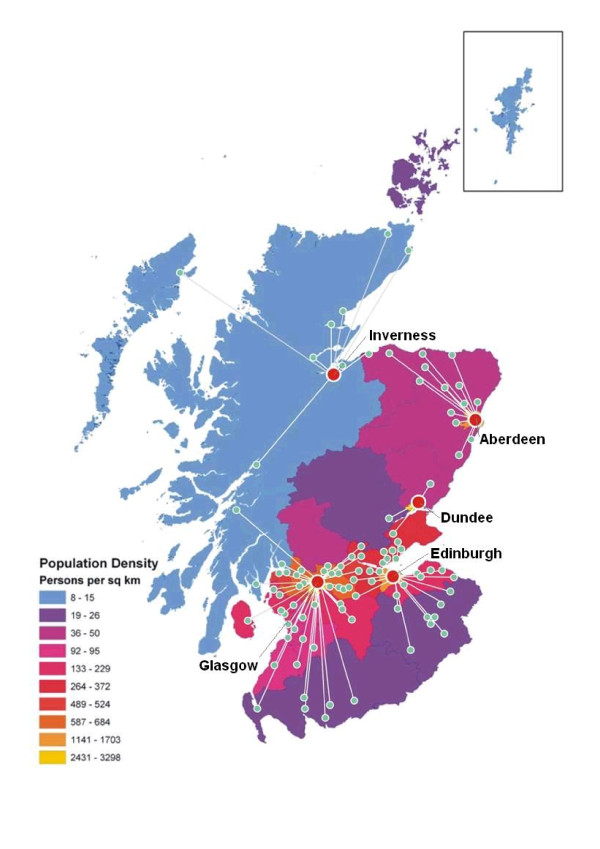
**GS:3D Locations**. Map of Scotland showing locations in which participants were recruited to GS:3D. SNBTS Donor Centres in Inverness, Aberdeen, Dundee, Edinburgh and Glasgow are indicated by large circles and clinics under the management of each centre are indicated by smaller circles with connecting lines.

Participants were recruited through the local management of SNBTS Donor Centres in Aberdeen, Dundee, Edinburgh, Glasgow and Inverness (represented by large circles in Figure 2). A wide geographic range was sampled by targeting recruitment to mobile clinics, as well as the Donor Centres. The study recruited participants at a total of 144 different sites, the locations of which are illustrated schematically in Figure 2. Where possible, an additional donor care assistant was provided for each clinic in which recruitment took place, funded through the award for the project. Statistics were not gathered as to the percentage of donors who agreed to participate in the research, but this proved to be ample for the requirements of the study, with recruitment completed within a period of seven months.

### Collection and processing of consent forms and demographic data

**Figure 3:**
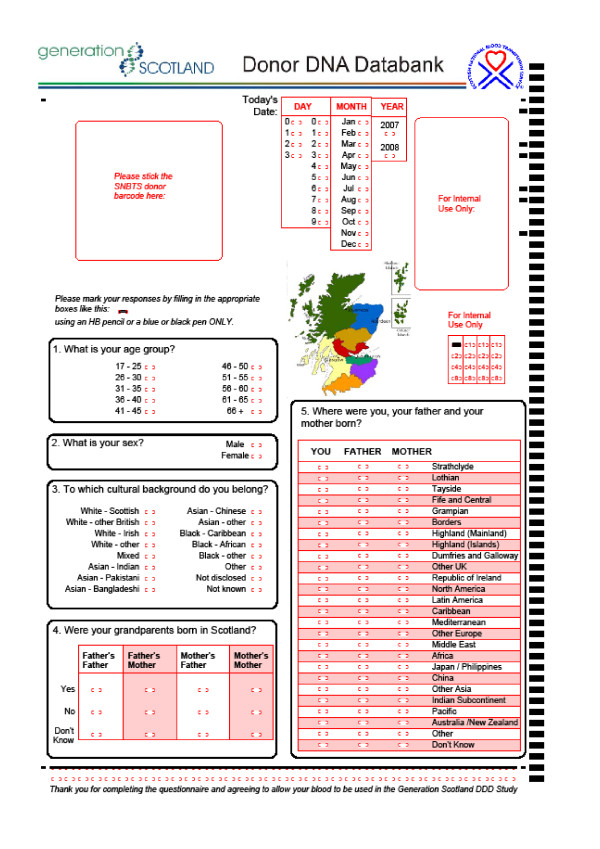
**GS:3D Questionnaire**. The Optical Mark Read Questionnaire.

Following introduction to the study by means of a publicity poster and leaflet, supplied upon attendance at a blood donation session, individuals who agreed to consider taking part were given a copy of the Participant Information Leaflet (PIL), a consent form, and a questionnaire [10]. The consent form was discussed and completed prior to any sample or phenotypic details being taken. Participants were informed that they were free to withdraw from the study during the following 28 days. Participants then returned the completed questionnaire (at which time there was an opportunity to clarify any matters arising with an SNBTS nurse), and gave permission for a blood sample to be used for research, all according to Standard Operating Procedures. Data were entered by each participant on a paper optical mark read (OMR) questionnaire, illustrated in Figure 3. The collection of data aimed to maximise the research benefit of each individual's participation while minimising inconvenience to the donor and disruption to the work of the National Health Service.

The questionnaires and attached consent forms from each session (up to 30 participants) were posted to the central research office in Edinburgh. On arrival, unique research ID labels corresponding to the appropriate paired filter and blood samples (see below) were added to the questionnaire and consent forms. Questionnaire data were entered on to a secure database by OMR scanning with a DRS Photoscribe PS900 IM2 scanner, which included a scan of the research ID barcode.

### Processing of leucodepletion filters and blood samples

As part of the routine processing procedure of volunteer blood donations in the U.K., blood is filtered to remove cells, and the used filters are an excellent source of DNA [11]. Leucodepletion filters identified as coming from study participants and containing cells for DNA extraction were sent from the SNBTS process and testing laboratories to the research laboratory (WTCRF Genetics Core, University of Edinburgh) in batches and stored for up to seven days prior to extraction. Whole blood samples taken from the prefiltration pouch were also received by the research laboratory in 9 ml EDTA (Becton Dickinson) tubes and stored at room temperature for up to five days from the date of collection until plasma was purified and stored. Although some plasma analytes are unstable over this period of time, many epidemiologically useful measurements can reliably be made [12]. Filters and EDTA tubes were physically paired up by matching SNBTS barcodes. A unique GS:3D research sample ID was then assigned to each SNBTS barcode. Paired samples were logged by scanning barcodes into the GS:3D database, a bespoke study management program. The GS:3D sample ID was subsequently entered into a Starlims Laboratory Information Management System (LIMS), which assigned the required number of LIMS barcode labels and storage space for the resulting processed sample aliquots of DNA and plasma. Use of a LIMS in an SOP-driven core laboratory operating to a GLP standard helps to minimise the risk of sample mix-up.

### Extraction and storage of DNA

The initial steps of the protocol for extracting DNA from the leucodepletion filters followed the method described by Cook *et al*, 2003 [11]. All filters underwent a "draw-through" step to wash out and collect leucocytes. This involved cutting the inlet and outlet tubes at either side of the filter and dispensing 20 ml of phosphate buffered saline (PBS) (GIBCO) pH7.4 through the filter in the counter-direction to that indicated by the arrow on the side of the filter. PBS was dispensed using a 30 ml Terumo syringe (TERUSS-30E5Z1) and 20 ml of draw-through was collected in a labelled 50 ml falcon tube with screw lid (Greiner Bio-one Ltd) and stored at -40°C until ready for extraction. As a quality control procedure, 100 μl of blood cells from the 20 ml sample were spotted onto an FTA nucleic acid capture card (Whatman) which was archived at room temperature in a secure store. This is designed to allow investigation of any sample mix-up during the process of DNA extraction.

Each 20 ml sample was split into two samples of 10 ml each for DNA extraction using a Nucleon Kit (Tepnel Life Science) with the BACC3 protocol. At the DNA precipitation stage, both the upper phases from the two corresponding DNA extractions (originating from the same filter) were layered into a 15 ml EZ Flip tube. The precipitated DNA was hooked out and placed directly into a labelled 2.0 ml microtube (Scientific Specialities Inc) containing 1.5 ml TE buffer pH 7.5 (10 mM Tris-Cl pH 7.5, 1 mM EDTA pH 8.0). Microtubes were rotated for 2 weeks at room temperature until DNA was fully re-suspended. 8 out of every batch of 92 samples were electrophoresed on a 1% agarose gel to test for integrity of the DNA, and all were satisfactory. DNA concentrations (ng/μl) and levels of protein and RNA contamination were determined for all samples using the NanoDrop method (Thermo Scientific). 500 μl of each DNA master stock were transferred to a deep well plate then normalised to 50 ng/μl to make working stock plates. The remaining 1000 μl were archived in a microtube at -40°C.

### Purification and storage of plasma

Whole blood in EDTA tubes was centrifuged for 15 minutes at 2000 g to separate plasma. Two aliquots of 1 ml of plasma were dispensed into 2 × 1.4 ml tubes (Fluid X Robo-rack-96) and labelled with a printed LIMS label and barcode. Pierceable lids (TPE Capclusters) were fitted to the tubes which were stored at -80°C in 96 position racks.

## Results

### Recruitment

A total of 5,934 participants were recruited to the study by SNBTS donor services staff. However, leucodepletion filters were not received from some participants, and these individuals were excluded as they were of no value to the study. Filters were not received by the research laboratory for a variety of reasons. These include the participant being enrolled in the study but then failing to provide a full donation of blood; the identifying marker on the filter not being noticed by the SNBTS laboratory and therefore not being diverted into the onward transport protocol after processing; or the blood failing one of the many sensitive safety tests routinely performed on it by the SNBTS.

A total of 5,230 filters which passed all tests were received by the University of Edinburgh WTCRF research lab. However, the corresponding consent and questionnaire forms were not received for every filter. This occurred for a variety of reasons including a single batch of 22 forms being lost in transit. All consents received were checked by the research team, and some were found to be invalid (unsigned or undated, etc), which meant that the participant had to be removed from the study. A small number of samples were destroyed due to problems during processing in the research lab. Together, this resulted in a total of exactly 5,000 participants with a complete set of data, valid consent and filter processed to cells ready for DNA extraction. Two of these participants withdrew from the study, after leaving the clinic and having time to reflect.

### Questionnaire data

**Figure 4:**
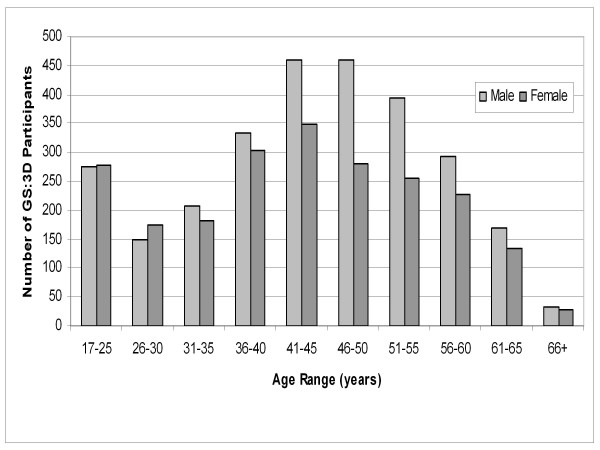
**Age Range and Sex of GS:3D participants**. Chart showing age range and sex of GS:3D participants. Males are represented by pale bars and females by dark bars.

The questionnaire (Figure 3) collected data on age group, sex, cultural origin, and place of birth of the participant, parents and grandparents. The forms were filled in well by participants. For example, Question 2, "What is your sex?" gave responses from the 4,998 study participants of 2,209 female, 2,775 male, 1 multiple marks and 13 missing marks. The age range and sex of the participants is illustrated schematically in Figure 4.

Over 88% of participants recorded their ethnicity as "White - Scottish", and 98% were in one of the four "White" categories (Table 1). This ethnicity pattern was similar to that reported in the Census of Population for Scotland, 2001, General Register Office for Scotland, (c) Crown copyright 2003. There is an under-representation of participants with "Asian" categories of ethnicity (0.12% in GS:3D and 1.41% in the Census) as is found for blood donors in general. 71% of participants had either three or four grandparents born in Scotland.

**Table 1:** Recorded ethnicity of GS:3D participants

Ethnicity	% Female	% Male	All GS:3D (%)	2001 Census
White - Scottish	87.66	88.95	88.38	88.09

White - Other British	10.07	8.95	9.45	7.38

White - Irish	1.04	0.90	0.96	0.98

White - Other	0.77	0.69	0.72	1.54

Mixed	0.36	0.25	0.30	0.25

Asian - Indian	0.00	0.11	0.06	0.30

Asian - Pakistani	0.00	0.00	0.00	0.63

Asian - Bangladeshi	0.00	0.00	0.00	0.04

Asian - Chinese	0.05	0.07	0.06	0.32

Asian - Other	0.00	0.00	0.00	0.12

Black - Caribbean	0.00	0.00	0.00	0.04

Black - African	0.00	0.00	0.00	0.10

Black - Other	0.00	0.00	0.00	0.02

Other	0.05	0.07	0.06	0.19

### Statistical power

The primary objective of this study was to establish a collection of samples and an accompanying database of demographic data as a resource for future analysis. Association studies, particularly genome-wide association studies using high density microarrays, are currently a widely used design for investigating the genetics underlying complex diseases [5,6]. This approach looks for a statistical association between genetic variants (SNP or copy number variants) and a defined phenotype [2]. An association study is usually conducted in a population-based sample of unrelated affected and unaffected individuals (a case-control study). The selection of appropriate and sufficient control samples is therefore crucial for success [5,7]. Ideally, control samples should reflect the ethnic and geographic (as a proxy for genetic) composition of case samples, and blood donors have been shown to be an appropriate control group for many complex conditions [5,6]. The statistical power of case-control studies can be increased by increasing the numbers of controls.

### Data and sample extraction and access

The GS:3D DNA samples all passed the routine quality control tests of intactness and purity described in Methods. While a few samples had low yield (less than 30 μg), the vast majority yielded over 1 mg of purified DNA. This is a significant amount of material, as each Taqman SNP genotype assay consumes up to 20 ng of DNA, and each Illumina whole genome scan (of up to 1 million markers) consumes around 200 ng of DNA. The GS:3D resource therefore should have an extremely long lifespan. If necessary, at some future point sample stocks could be replenished through the technique of whole genome amplification [13].

Genotype data from analysis of the DNA samples is at an early stage, but initial results show the first few hundred samples to be of good quality with a high call rate in Taqman SNP genotyping assays (L. Murphy, WTCRF, pers. comm.). Master and working stocks of DNA and aliquots of plasma are available to researchers in the U.K. for hypothesis-driven analyses with appropriate ethical approval. An access process has been defined with reference to Wellcome Trust and Medical Research Council guidance [14]. Access to the samples and data is reviewed by the Generation Scotland Resource Management and Development Committee, and only de-identified data can be made available. Researchers will be obliged to return derived data after an agreed time period, and acknowledge the resource in publications arising.

### IT infrastructure

The development of an IT infrastructure that can support genetics research is an important part of Generation Scotland. Innovative aspects of the IT infrastructure in GS:3D are primarily in the use of optical mark read (OMR) technology to collect questionnaire data and the development and implementation of web-based study management tools. A web-based interface provides password-protected access to summary statistics on the questionnaire data. Participants are identified by a unique research ID number, the production and usage of which is tightly monitored. Systems were developed to ensure efficient and confidential handling and management of all data and samples. The GS:3D database was used to record all exceptions (e.g. consent form not fully filled in, no tube received for plasma), resulting in a complete audit trail of all samples and data that were part of the study. These study management systems are outlined in Macleod *et al*, 2009 [9]. In due course a cumulative genotype database will be available to researchers through a web portal with appropriate permissions and security. Genotype data across the whole set of DNA samples, ideally genome wide at high density, should be available in the future.

### Ethical issues

All components of GS:3D, including the protocol and written materials provided to participants, have received ethical approval from the NHS Research Ethics Committee for Scotland A (REC reference number: 06/MRE00/105). In addition, local approval has been obtained from NHS Lothian, Glasgow and North of Scotland Research Ethics Committees, from NHS Research and Development Offices, and from an SNBTS management committee. GS:3D has been granted Research Tissue Bank status by the Tayside Committee on Medical Research Ethics B (REC Reference Number: 10/S1402/21).

The study aimed to minimise several risks to participants:

1. The physical and administrative separation of the clinical (SNBTS) and research (University of Edinburgh) teams was designed to minimise the risk to the privacy of participants, while maximising rate of recruitment.

2. No personal or identifier information was given to the research team by the SNBTS and there is no mention of participation in the study on the donor service record. After a period of 28 days during which participants were able to withdraw, all links to the SNBTS identifier were broken.

3. It is impossible to know what precise purposes the resource will be used for in the future; therefore fully informed consent for the use of data and DNA and plasma samples cannot be obtained. Instead, "blanket" consent was sought, with ethics approval through the research tissue bank access process required for each new use.

4. It was emphasised that participation was entirely voluntary. Withdrawal was allowed up to 28 days after the donation session, upon which all data and samples relating to the withdrawing participant were destroyed. After the 28 day period, withdrawal was not possible because the samples and data were de-identified.

## Discussion

GS:3D will be of particular utility to studies of Caucasian populations, but should also have wider applications, for example in testing new genotyping methodologies. The resource is complementary to other population-based genetic epidemiology studies, such as the Generation Scotland: Scottish Family Health Study [8] and the UK Biobank [15], which were established primarily to characterise genes and genetic risk in the population. The model of recruitment described here differs from that of most genetic cohort studies by using the infrastructure and expertise of the Blood Transfusion Service. Other study designs usually involve research clinics, which have the advantage of allowing more study-specific data to be collected, but the disadvantage of significantly increased recruitment costs. Such models of recruitment require funding at the level of programme grants, rather than the project grant which was sufficient to implement GS:3D. Although detailed phenotyping of traits relevant to complex diseases is not available in GS:3D, inclusion of participants who would meet the criteria for a case is likely to be rare, due to the stringent blood donor exclusion criteria described in the Methods section. Furthermore, low levels of such misclassification should only have a small adverse effect on power [7]. The Wellcome Trust Case-Control Consortium initially used a control:case ratio of 1.5:1, combining 1958 Birth Cohort and UK Blood Transfusion Service controls, but also expanded this to up to 7.5:1 by including cases for other diseases as controls. This expansion increased evidence for association at most of the loci that received the strongest support from the primary analysis [5].

## Conclusions

The GS:3D study protocol has allowed the efficient generation of a new large scale resource of DNA and plasma samples. This collection is suitable for use in genetic studies of human disease and the sample size is large enough to give substantial numbers of controls selected on the basis of age range, gender, ethnic or geographic origin. The use of blood donors is a cost-effective way to collect a large number of control DNA samples. The availability of the GS:3D resource should reduce costs to investigators who would otherwise have had to recruit their own controls.

## Competing interests

The authors declare that they have no competing interests.

## Authors' contributions

All authors contributed to the writing of the manuscript, in an iterative manner. SK was the study co-ordinator and project manager. DCML and AC designed and implemented the IT systems used to manage the study data and samples. KT performed the laboratory work to extract and characterise DNA and plasma from blood. SW and DN provided clinical and epidemiological advice and expertise. MT was Chief Investigator and led the clinical aspects of the study. DP was Principal Investigator, conceived the study and led the scientific aspects. The main text was written by the study co-ordinator SK, with comments and amendments made by all authors, who have each read and approved the final manuscript.

## Pre-publication history

The pre-publication history for this paper can be accessed here:

http://www.biomedcentral.com/1471-2350/11/166/prepub
